# Influence of Strain Hardening Rate of Material on Temperature and Strain Distributions during Wire Drawing

**DOI:** 10.3390/ma16145203

**Published:** 2023-07-24

**Authors:** Joong-Ki Hwang

**Affiliations:** School of Mechatronics Engineering, Korea University of Technology & Education, Cheonan 31253, Republic of Korea; jkhwang@koreatech.ac.kr; Tel.: +82-041-560-1642

**Keywords:** strain hardening exponent, wire drawing, temperature increase, strain distribution

## Abstract

Temperature rise of a specimen is a significant issue in drawing industries for wire, rod, and bar products, because an excessive increase in temperature during the drawing process can deteriorate the product quality and die life. The influence of the strain hardening exponent (*n*) of a wire on the temperature and strain distributions during wire drawing is investigated to understand its effect and to improve the quality of drawn wire. Finite element analysis and experiments are conducted to analyze the temperature and strain distributions of wires with *n* values of 0.0, 0.1, 0.5, and 1.0. The temperature increase of the wire augments as the *n* of the wire increases, despite the same amount of ideal plastic deformation, which is associated closely with the redundant work. The shear strain increases with the *n* of the specimen, which generates redundant work, leading to a high temperature rise. Similarly, drawing force increases with the *n* of the specimen, owing to the increase in redundant work with the *n* of the wire. In addition, the drawing force presents a linear relationship with the temperature rise of the wire. The drawing speed should be reduced and/or the cooling of wire and die should be strengthened during wire drawing, with increasing *n* value of the wire, because product quality and die wear are highly associated with the temperature rise of the wire in the deformation zone.

## 1. Introduction

The temperature control of both the workpiece and tool is one of the prime issues in the cold metal-forming process, because an excessive increase in temperature during the forming process can deteriorate the product quality and tool life. In wire-drawing industries for wire, rod, and bar products, an increase in temperature during the process is a significant issue, as the drawing speed increases owing to high productivity [1,2]. For example, in plain carbon steels, temperature increase deteriorates the ductility and drawability of the wire during wire drawing because of the strain aging effect [3,4]. Thus, drawing speed or productivity of plain carbon steels, particularly pearlitic steels, can only be increased to a certain extent, owing to the temperature rise during wire drawing.

In wire drawing, the temperature increase of a specimen is determined by the process conditions, such as the drawing speed (*V_d_*), reduction in area per pass (*R_p_*), friction coefficient between the specimen and die (m), and semi-die angle (*θ*), as well as material properties, such as the thermal conductivity (*k*), heat capacity (*C_p_*), density (*ρ*), and strain hardening exponent (*n*) of a specimen. The effects of process conditions on the temperature increase of materials have been extensively investigated over the past five decades [3,4,5,6,7,8,9,10,11,12]. For example, researchers reported that an increase in *V_d_*, *R_p_*, and m increased the temperature of a specimen. In the case of *θ*, an optimum value of *θ* resulted in minimal temperature increase. 

**Figure 1:**
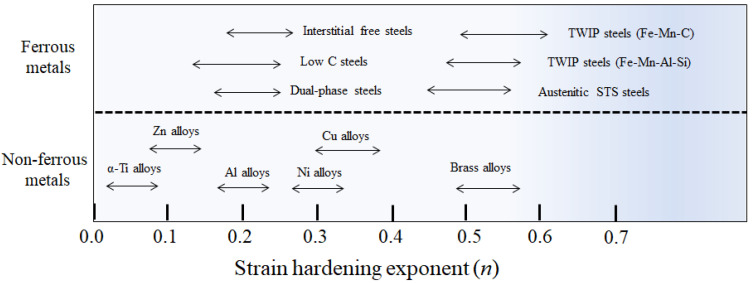
Schematic illustration of *n* values of various ferrous and non-ferrous metals; adapted from [16,17,18,19,20,21,22,23,24,25].

However, studies regarding the effects of material properties on the temperature increase of a material during wire drawing are insufficient. That is, studies regarding the influence of *n* of a material on the temperature increase during wire drawing are few [13], although several studies have been performed to investigate the effect of *n* on the strain distribution during wire drawing [14,15]. Figure 1 summarizes the *n* values of typical metals from the literature [16,17,18,19,20,21,22,23,24,25]. The *n* values of metals, including wire rod products, vary significantly, from 0.1 to 0.6. The *n* of wire rod products has increased because wire rod industries are developing new products in the direction of increasing both the strength and formability. For example, the automobile industry intends to manufacture products that reduce car weight by increasing strength and the elimination of expensive heat treatments, via an improvement in formability. In the same vein, Hwang et al. [26] recommended using twinning-induced plasticity (TWIP) steel for wire, rod, and bar products, such as bearings and fasteners, because TWIP steels offer an excellent combination of strength, ductility, and toughness, which is due to their high *n* values (Figure 1) arising from mechanical twinning and dynamic strain aging during plastic deformation [27,28]. The behavior of temperature increase in TWIP steels may differ from that of plain carbon steels or non-ferrous metals, owing to their high *n* values. The *n* of TWIP steels can be more than twice that of plain carbon steels and non-ferrous metals, as shown in Figure 1; thus, TWIP steels and plain carbon steels exhibit different thermal behavior during wire drawing. Even under the same drawing conditions, the *V_d_* and/or cooling condition should be changed with the material, especially with *n*, because the temperature distribution of a specimen is different during the drawing process depending on the material properties. Therefore, it is industrially important to understand the thermal behavior of a specimen during the drawing process according to the *n* value of a wire, to find the optimum drawing condition. However, studies regarding the effect of the *n* of a metal on its temperature increase during wire drawing are few. In particular, to the best of the author’s knowledge, no comprehensive study was reported on the correlation between the *n* value and temperature increase of the specimen during wire, rod, and bar drawing. 

Hence, the effect of the *n* of a metal on the temperature increase during wire drawing should be investigated to elucidate the wire-drawing behavior in more detail and improve the quality of drawn wire. In the present study, the effect of the *n* of a wire on the temperature increase and strain distribution is analyzed during wire drawing. Finite element analysis (FEA) and experiments were performed to analyze the temperature and strain distributions of wires with *n* values of 0.0, 0.1, 0.5, and 1.0. 

## 2. Experiment and FEA

### 2.1. Experiment

**Figure 2:**
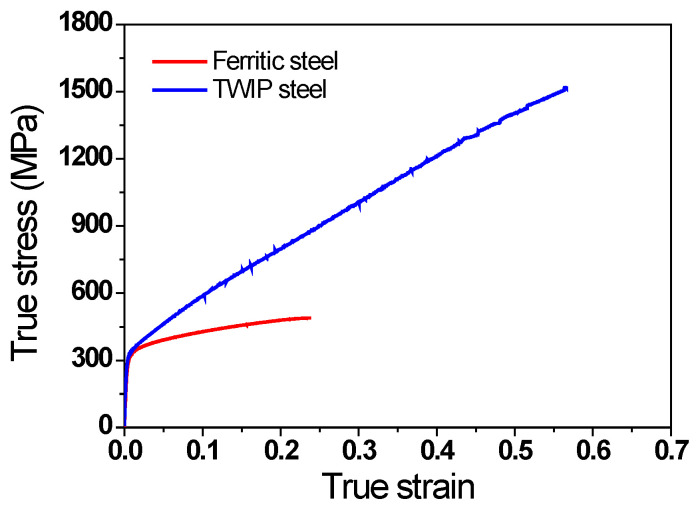
Comparison of true stress–strain curves of ferritic and TWIP steels used in current study.

To validate the current FEA model, wire-drawing tests were conducted on ferritic steel with low *n* and TWIP steel with high *n* values using a single pass-type draw-bench machine. The 13 mm diameter ferritic steel wire rod used was provided by steel-making company, POSCO, in South Korea. This wire rod was manufactured by heating the billet with a 160-mm-wide square shape at approximately 1200 °C, followed by performing normal hot rolling at temperature ranges of 900–1200 °C, and Stelmor air cooling at a cooling rate of approximately 3 °C/s [29]. The chemical composition of the ferritic steel was Fe-0.1C-0.4Mn-0.1Si (wt.%). For the case of TWIP steel, a 50 kg ingot with a chemical composition of Fe-0.72C-17.07Mn-2.9Cu (wt.%) was cast via induction melting under Ar gas. To reduce Mn segregation in the ingot, the ingot was soaked at 1200 °C for 12 h, and then rolled down to a plate measuring 20 mm thick, at a final rolling temperature of 950 °C, followed by air cooling at 21 °C. For the tensile test, the specimens were extracted along the rolling direction from the hot-rolled ferritic steel rod and hot-rolled TWIP steel plate. Subsequently, the specimens were machined into a tensile specimen with a gauge diameter of 5.0 mm and length of 25.0 mm, using a lathe. The specimens were assumed to be isotropic materials because hot-rolled steels were used in this test. The specimens were strained at a low strain rate of 10^−3^ s^−1^ using an Instron at a room temperature (RT) of 26 °C. Figure 2 shows the true stress–strain curve of the two specimens. Based on the curve fitting of the two true stress–strain profiles using Hollomon’s law, the *n* value of ferritic steel was 0.16 and that of TWIP steel was 0.53. 

For the wire-drawing test, round rods with a 13 mm diameter were prepared using lathe from the TWIP steel plate along the rolling axis. The 13 mm diameter wire rod was drawn into an 11.63 mm diameter wire with a *V_d_* of 0.07 m/s using the single die-type draw-bench at RT. The process conditions of wire-drawing test were summarized in Table 1. Prior to the test, the oxidation scale on the specimen was removed via chemical pickling using 12.5% HCl solution. MoS_2_ lubricant was sprayed onto the specimen. *θ* was 6°, and *R_p_* was approximately 20%. *R_p_* was calculated as follows:

(1) $$R_{p}=\frac{d_{0}^{2}-d_{f}^{2}}{d_{0}^{2}}\times 100\;\left(\%\right)$$

where *d*_0_ and *d_f_* are the diameters of initial and drawn wires, respectively. The nominal drawing strain (*ε_n_*) of the drawn wire was 0.22, which was calculated as follows:

(2) $$\varepsilon_{n}=2ln\frac{d_{0}}{d_{f}}$$

The temperature in the core region of the specimen was measured using a K-type thermocouple with 1.0 mm diameter. To reduce temperature disturbances at the surface of the wire, a thermocouple was embedded in a 1.0 mm diameter hole in the wire [12,29]. Additionally, a load cell installed on the draw-bench machine was used to measure the drawing force.

### 2.2. FEA

During a typical wire drawing, the temperature of the wire increases from heat generation owing to the deformation of the wire, as well as the friction between the wire and die interface [30]. Consequently, the surface region of the wire exhibits a higher temperature rise than the center region, owing to heating caused by friction at the wire and die interface. Unfortunately, measuring the surface temperature of round-shaped small specimens was difficult using both non-contact-type pyrometers and contact-type thermocouples [29]. Furthermore, a wire experiences inhomogeneous plastic deformation along its radial direction depending on operating conditions during wire drawing [31,32,33], thus resulting in a complex temperature distribution within the wire. Hence, FEA was applied in this study to evaluate both the temperature and strain distributions of the specimen, because FEA is an effective approach for analyzing the complex temperature, strain, and stress distributions in deformed specimens. The DEFORM commercial software (version 11.0) was used in simulating the wire-drawing process. Based on the experiences of the author, this FEA software well-simulates bulk-forming processes, such as forging, wire drawing, and rolling; therefore, it is suitable for analyzing the wire-drawing process. 

The die was considered as a rigid body, i.e., it did not deform during the entire drawing process. The die angle was 6°. The shear friction factor was set as 0.1765, based on a previous FEA [34]. The initial wire with the diameter of 13 mm was drawn into wire with the diameter of 11.63 mm at a velocity of 0.07 m/s at RT. Half of the entire geometry was modeled stemming from the symmetric natures of the wire-drawing process, and approximately 5000 square elements were used in wire specimen. 

During plastic deformation, the temperature rise was numerically obtained as follows [35]:

(3) $$\Delta T_{i}=\frac{\Delta Q}{\rho C_{p}}=\frac{\xi }{\rho C_{p}}\int_{0}^{\varepsilon_{1}}\sigma d\varepsilon$$

where *T_i_* and *Q* are the ideal temperature increase and heat energy, respectively; and *ξ* is a fraction factor representing the ratio of *Q* to mechanical work. In this FEA, *ξ* was assumed to be 0.9 because only a slight amount of mechanical energy was stored in the deformed specimen as elastic energy [35,36]. The thermal properties of the specimen and die, as listed in Table 2, were assumed to be unaffected by temperature because the temperature variation in the wire and die was small; namely, 25–150 °C.

The required flow stress curves for the FEA were obtained by analyzing *T_i_* during plastic deformation using Equation (3). The specimen was considered as an isotropic material; thus, the wire’s constitutive behavior can be described by Hollomon’s law using the strain hardening coefficient (*K*) and *n* as follows:

(4) $$\sigma =K\varepsilon^{n}$$

**Figure 3:**
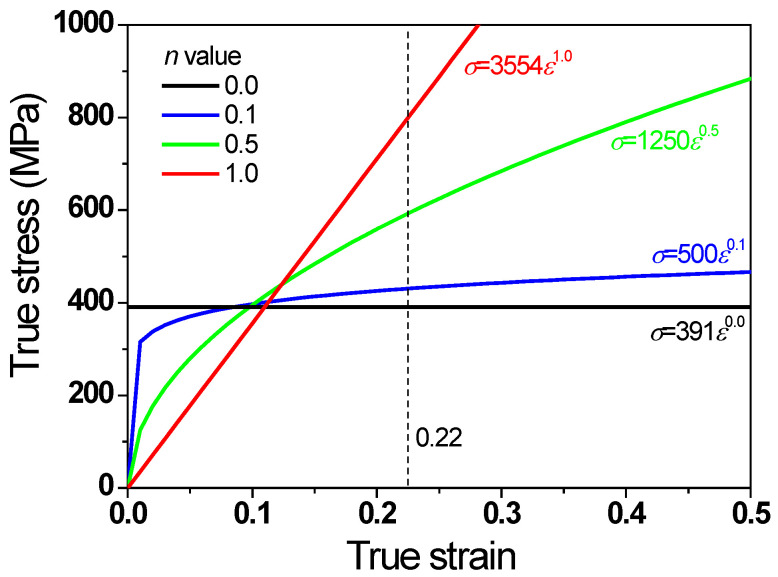
Comparison of flow stress of metals with different *n* values.

In this case, Δ*T_i_* during plastic deformation was easily calculated as follows:

(5) $$\Delta T_{i}=\frac{\xi }{\rho C_{p}}\int_{0}^{\varepsilon_{1}}K\varepsilon^{n}d\varepsilon =\frac{\xi }{\rho C_{p}}K\frac{\epsilon_{1}^{n+1}}{n+1}$$

where *ε*_1_ was defined to be 0.22 based on Equation (2). The values of *K* were calculated for fixed *n* values of 0.0, 0.1, 0.5, and 1.0 under the assumption of a constant $\Delta T_{i}$. The flow stresses of the metals are shown in Figure 3 and Table 3. For convenience, wire with *n* values of 0.0, 0.1, 0.5, and 1.0 is referred to as non-hardening, low-hardening, high-hardening, and linear-hardening wires, respectively.

## 3. Validation of FEA Model

**Figure 4:**
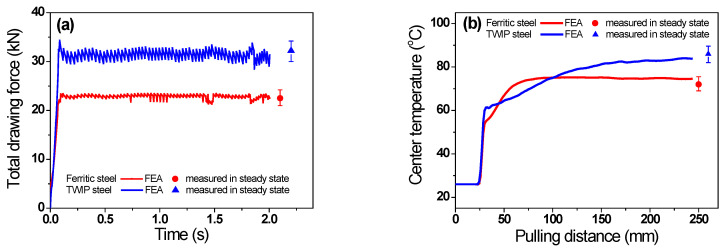
Comparison of equilibrium (**a**) total drawing force and (**b**) center temperature of two steels between experimental and FEA results.

Before analyzing the FEA results, the accuracy of the current FEA model was verified by comparing the simulated and measured drawing forces and temperatures of the two steels. Figure 4a shows a comparison of the total drawing forces (*F_t_*) of the two steels. The simulated *F_t_* was consistent with the measured *F_t_*. The *F_t_* of the TWIP steel was higher than that of the ferritic steel. Figure 4b compares the equilibrium temperature (*T*_eq_) calculated via FEA and that measured using the thermocouple. The simulated *T*_eq_ of the wire was in agreement with the measured *T*_eq_. The temperature increase of the two steels showed a pattern similar to that of *F_t_*. The *T*_eq_ of the TWIP steel was higher compared with that of the ferritic steel. Based on the validation of *F_t_* and *T*_eq_, one can conclude that the results of the present FEA are acceptable and reliable for further analysis.

## 4. Results

### 4.1. Temperature Distribution

**Figure 5:**
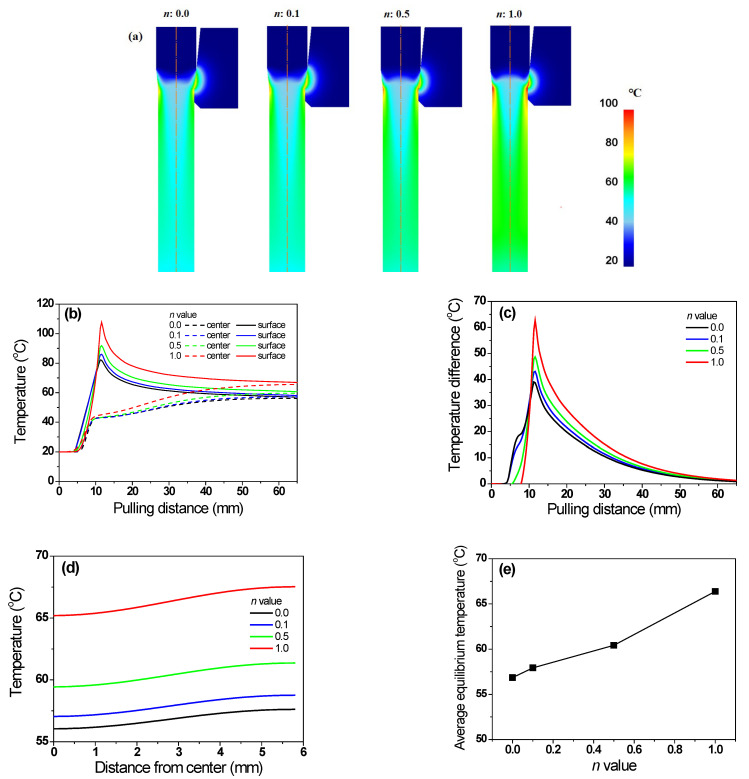
Comparison of temperature (**a**) contours and (**b**) profiles along drawing direction at center and surface regions, and (**c**) temperature difference between center and surface regions of specimens with different *n* values; (**d**) comparison of temperature profiles along radial direction of specimen at 50 mm from die exit and (**e**) average temperature with different *n* values.

Figure 5a shows the numerical simulation results for the temperature distribution of the specimens with different *n* values. Figure 5b compares temperature profiles along the drawing direction at the center and surface regions of the specimen. Regardless of the *n* of the specimen, the maximum temperature was recorded in the surface region of the wire at the inlet of the die’s bearing zone. The maximum temperature increased with the *n* of the specimen, which is consistent with the previous results of El-Domiaty and Kassab [13]. A temperature gradient existed along the radial direction of the wire, because frictional heating at the surface region led to a steep temperature rise at the surface region. In all steels, the center temperature (*T_c_*) increased gradually; whereas, the surface temperature (*T_s_*) increased rapidly and then decreased gradually, because most of the heat generated by friction was transferred to the specimen interior via conduction heat transfer, until the specimen temperatures reached equilibrium. Additionally, the ambient air cooled the surface of the specimen via convection and radiation heat transfers, although the influence was relatively insignificant. For example, in the high-hardening wire, the *T_c_* increased gradually from 42 °C to 62 °C; whereas, the *T*_s_ decreased rapidly from 93 °C to 62 °C. Figure 5c compares the temperature difference (*T_d_*) between the *T_c_* and *T_s_* of specimens with different *n* values. The *T_d_* was calculated as follows:

(6) $$T_{d}=T_{s}-T_{c}$$

The *T_d_* increased with the *n* of the specimen. Figure 5d shows the comparison of temperature profiles of the wire along the radial direction at a location 50 mm from the die exit with different *n* values. The average temperature increased with the *n* of the specimen (Figure 5e).

### 4.2. Strain Distribution 

**Figure 6:**
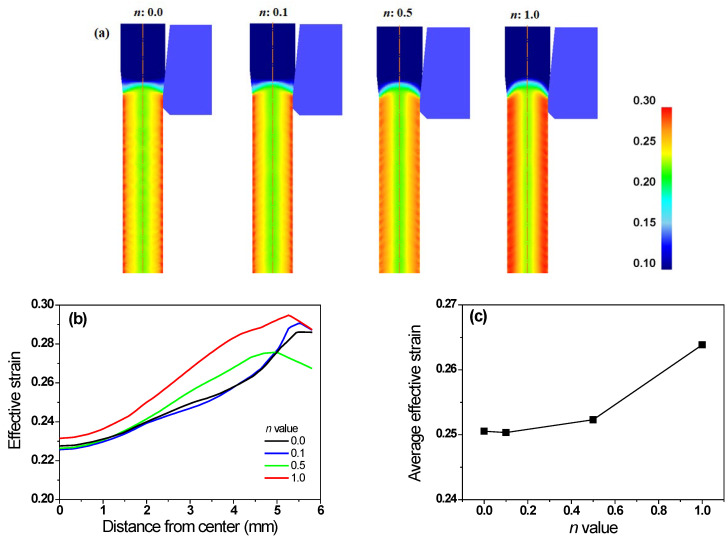
Comparison of effective strain (**a**) contours and (**b**) profiles along radial direction of specimens with different *n* values. (**c**) Variation in average effective strain as a function of *n* of wire.

Figure 6 shows the effective strain (*ε*_eff_) of the specimens with different *n* values. All wires exhibited the maximum *ε*_eff_ near the surface, and the minimum value at the center. This implies that the temperature increase in the surface region was higher than that in the center region, owing to the higher plastic deformation during wire drawing. The average *ε*_eff_ increased with the *n* of the specimen (Figure 6c).

**Figure 7:**
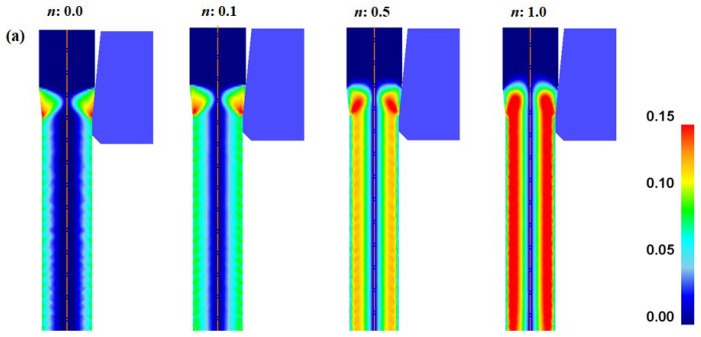

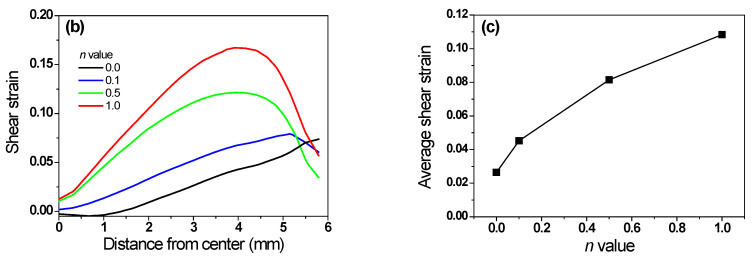
Comparison of shear strain (**a**) contours and (**b**) profiles along radial direction of specimens with different *n* values. (**c**) Variation in average shear strain as a function of *n* of wire.

Figure 7 compares the shear strains (*ε*_s_) of the specimens with different *n* values. The *ε*_s_ along the radial direction of the wire exhibited a trend similar to that of *ε*_eff_. In other words, *ε*_s_ increased and then decreased as the distance from the center increased, and *ε*_s_ was zero at the center region regardless of the *n*. Interestingly, the profiles of *ε*_s_ differed depending on the *n*. *ε*_s_ increased with increasing the *n* of the specimen. Accordingly, the non-hardening wire exhibited the highest uniformity along the radial direction of the wire.

**Figure 8:**
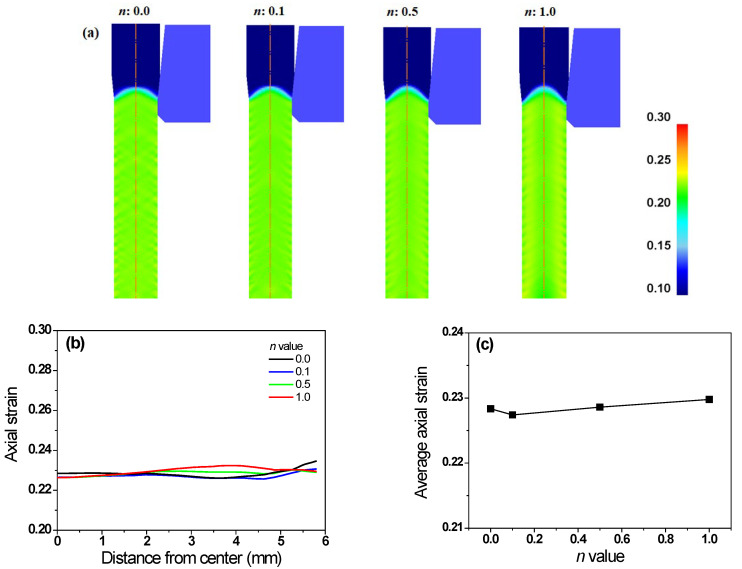
Comparison of axial strain (**a**) contours and (**b**) profiles along radial direction of specimens with the different *n* values. (**c**) Variation in average axial strain as a function of *n* of wire.

Figure 8 shows a comparison of the axial strain (*ε*_axi_) of the wires with *n*. Regardless of the *n* of the specimen, the *ε*_axi_ was similar. Based on the analysis of *ε*_eff_, *ε*_s_, and *ε*_axi_, the different *ε*_eff_ of the specimens with *n* were associated closely with the distribution of *ε*_s_ with the *n* of the wire. 

### 4.3. Stress, Damage Value, and Drawing Force

**Figure 9:**
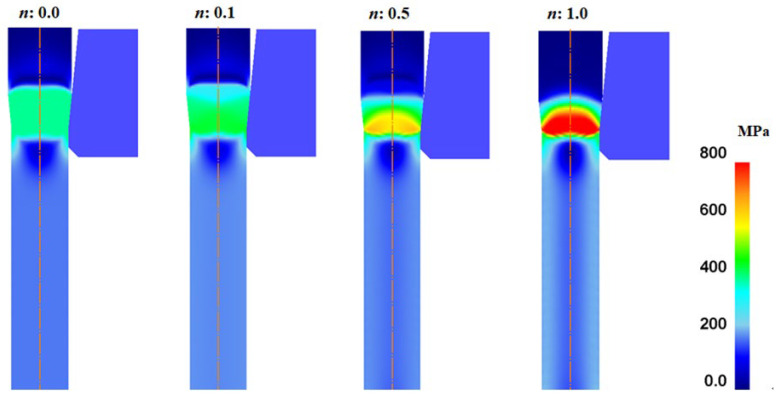
Comparison of effective stress contours of wire with different *n* values.

Figure 9 shows the effective stress (*σ_eff_*) of the specimens with the *n*. Regardless of the *n*, the maximum *σ_eff_* occurred in the surface region. Meanwhile, *σ_eff_* increased with the n of the specimen because of the higher strengthening of the wire with the applied strain as the *n* of the wire increased (Figure 3). 

**Figure 10:**
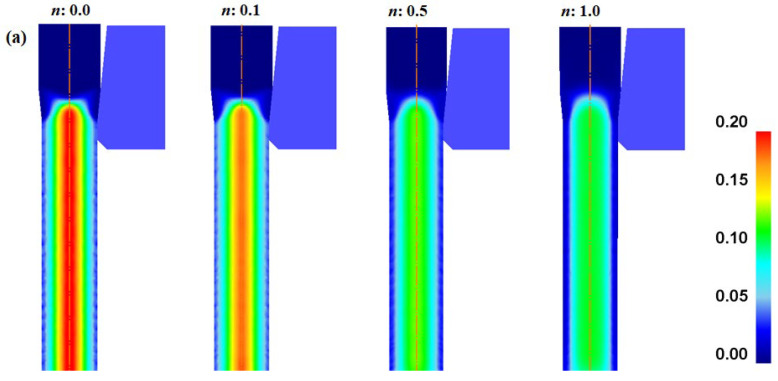

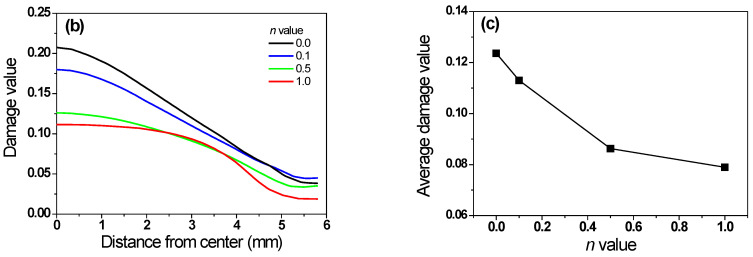
Comparison of damage value (**a**) contours and (**b**) profiles along radial direction of specimen with different *n* values. (**c**) Variation in average damage value as a function of *n* of wire.

The damage value of the wire (*D_w_*) was analyzed during the process to evaluate the formability with the *n* of the specimen. The normalized Cockcroft and Latham fracture criterion [39,40] shown below was applied:

(7) $$\;D_{w}=\int_{0}^{\varepsilon_{f}}\frac{\sigma_{m}}{\sigma_{eff}}d\sigma_{eff}$$

where *σ_m_* is the maximum tensile stress. Figure 10a compares the contours of *D_w_* for the drawn wire with the *n*. The distribution of *D_w_* varied with the *n*. The center region exhibited the maximum *D_w_* and the surface region exhibited the minimum value during the drawing process, which is consistent with the previous results of Cao et al. [41]. The maximum and average *D_w_* values decreased as the *n* of the specimen increased, as shown in Figure 10b,c, because *σ_eff_* increased with increasing *n* of the specimen (Figure 9). This implies that the non-hardening wire was easy to fracture in the center region during wire drawing. In other words, the probability of a central burst increased during wire drawing as the *n* of the specimen decreased. In fact, there are several reports regarding the central burst in metals with low *n* values, whereas there are no such reports in metals with high *n* values [42,43,44]. In addition, Haghighat and Parghazeh [45] reported that the probability of a central burst decreased with increasing the *n* of the specimen during rod extrusion. 

**Figure 11:**
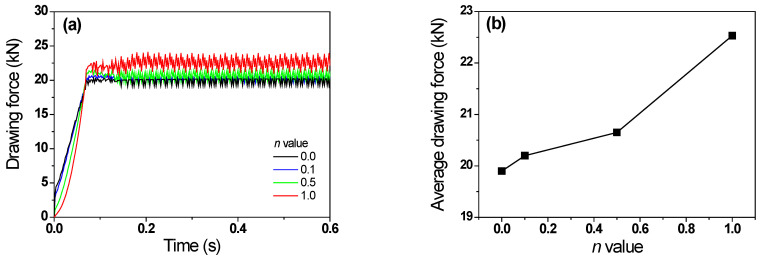
Comparison of (**a**) profiles and (**b**) average values of drawing force with different *n* values of wire.

**Figure 12:**
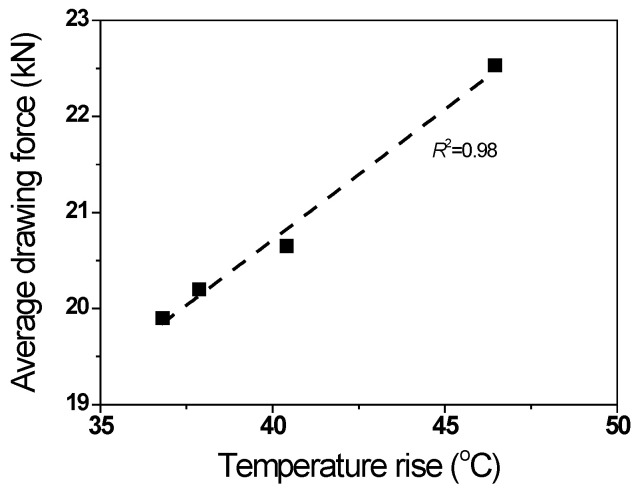
Relationship between drawing force and temperature increase during wire drawing.

Figure 11 shows a comparison of *F_t_* with the *n* of the specimen. *F_t_* increased with the n of the wire. The non-hardening wire exhibited the minimum *F_t_* of 19.8 kN, and the linear-hardening wire exhibited the maximum *F_t_* of 22.5 kN. *F_t_* showed a pattern similar to that of Δ*T_t_* with the *n*. Figure 12 shows the relationship between *F_t_* and Δ*T_t_* during wire drawing. Specifically, *F_t_* and Δ*T_t_* exhibited a linear relationship as follows:

(8) $$F_{t}=9.83+0.27\Delta T_{t},\;\;\;\;\;\;\;\;R^{2}=0.98$$

Vega et al. [7] suggested that *F_t_* varies linearly with the temperature rise of die based on the experimental drawing test, which is consistent with the present result.

## 5. Discussion

The most prominent result of this study is the higher Δ*T_t_*, *F_t_*, and *ε*_eff_ values of high-hardening wires, compared with those of low-hardening wires during wire drawing. In other words, Δ*T_t_*, *F_t_*, and *ε*_eff_ increased with the *n* of the wire. 

According to classical theory [46], the *F_t_* for wire drawing includes three factors: an ideal drawing force (*F_i_*) by the homogeneous deformation, an additional drawing force caused by the frictional effect (*F_f_*) at the specimen and die interface, and redundant effect (*F_r_*) owing to the die shape. Accordingly, *F_t_* can be calculated from the three aforementioned forces, as follows:

*F_t_* = *F_i_* + *F_f_* + *F_r_* (9)

**Figure 13:**
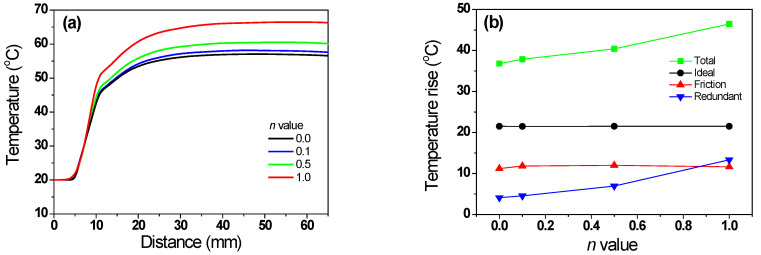

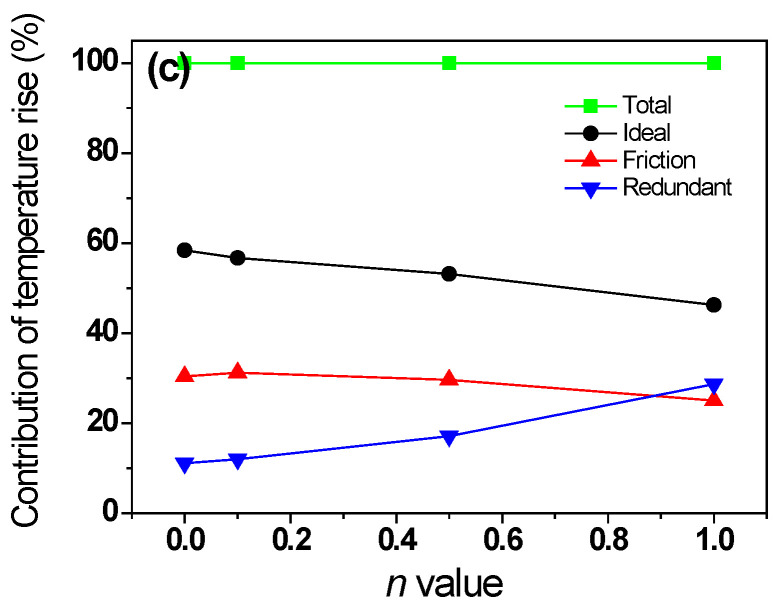
(**a**) Comparison of FEA results of quarter temperature of wires based on Figure 5a. Variations in (**b**) amount and (**c**) percentage of temperature increase of wire with *n* during wire drawing.

**Figure 14:**
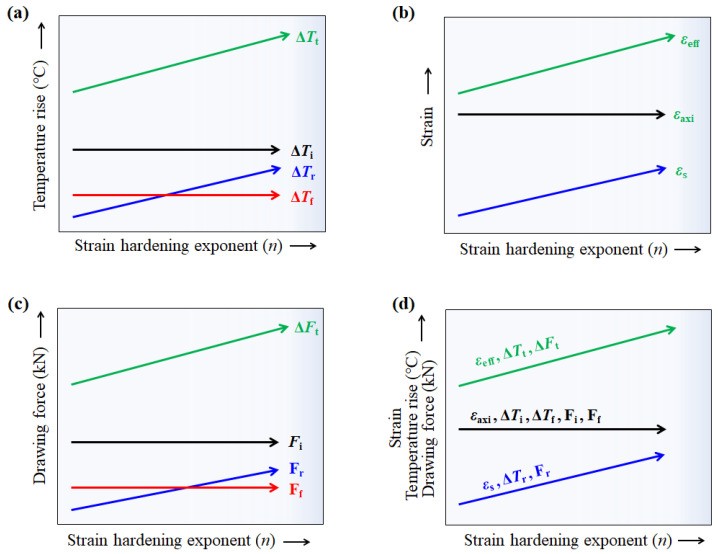
Schematic illustration showing effect of *n* of wire on (**a**) temperature increase, (**b**) strain, and (**c**) drawing force. (**d**) Schematic illustration showing effect of redundant strain with *n* of wire on wire-drawing behavior.

In addition, Δ*T_t_* comprises Δ*T_i_*, temperature increase caused by frictional work (Δ*T_f_*), and temperature increase caused by redundant work (Δ*T_r_*) as follows:

Δ*T_t_* = Δ*T_i_* + Δ*T_f_* + Δ*T_r_* (10)

where Δ*T_i_* was associated with *F_i_*; thus, Δ*T_i_* can be calculated using Equation (5). In this study, the flow stresses of the four specimens were designed to generate the same Δ*T_i_* regardless of the *n*; therefore, the initial increase in *T_c_* of all specimens was the same, as shown in Figure 5b. The Δ*T_r_* of the four specimens can be calculated using the profile of *ε*_eff_ (Figure 6b), because it can include the additional redundant work due to the conical die design. The Δ*T_r_* of the four specimens was obtained using Figure 6b and Equation (5). Finally, Δ*T_f_* was calculated using Equation (10). From the FEA, the average temperature can be defined as the temperature of the quarter region in the wire, and these temperature profiles were shown in Figure 13a with the *n* of the wire. Figure 13b compares the Δ*T_t_*, Δ*T_i_*, Δ*T_f_*, and Δ*T_r_* with the *n* of the specimen. As expected, Δ*T_i_* remained unchanged regardless of the wire. Additionally, Δ*T_f_* was similar for all four specimens. However, Δ*T_r_* increased with the *n* of the specimen. For example, the Δ*T_r_* of the non-hardening wire was 4.1 °C, which is 11.2% of Δ*T_t_*. By contrast, the Δ*T_r_* of the linear-hardening wire was 13.3 °C, which is 28.8% of Δ*T_t_*. Therefore, the increase in Δ*T_t_* of the specimen with the *n* is associated with redundant work, as schematically shown in Figure 14a. Notably, *ε*_s_ increased with the *n* of the specimen, as shown in Figure 14b and based on Figure 7, and this *ε*_s_ resulted in redundant work, leading to high Δ*T*_r_. 

Similarly, the increase in *F_t_* with the *n* of the specimen is associated closely with *F_r_* during wire drawing (Figure 14c). Figure 14d summarizes the influence of *ε*_s_ on the temperature rise and drawing force with the *n* of the wire. The increases in temperature and drawing force with the *n* of the wire were strongly related to *ε*_s_ during wire drawing, and not ε_axi_ because *ε*_axi_, Δ*T_i_*, Δ*T_f_, F_i_*, and *F_f_* remained almost constant regardless of the *n* of the wire. Finally, it should be noted that the drawing speed should be decreased with increasing *n* of the specimen, because the product quality, as well as die wear, is associated with the temperature increase of the wire in the deformation zone [3,47]. From the results of this study, engineers need to choose optimum drawing conditions based on the variation of the drawing force and wire temperature with process conditions and materials. However, it is not easy to present quantitative suggestions for engineers based on the results of this study. Currently, various studies are being conducted to find optimal process conditions using artificial intelligence [48,49]. Further research under more industrially specific conditions is required to design the optimum wire-drawing process with *n* value of material.

## 6. Conclusions

Temperature rise of a specimen is a significant issue in drawing industries for wire, rod, and bar products, because an excessive increase in temperature during the drawing process can deteriorate the product quality and die life. The effect of the *n* value of a wire on the temperature and strain distributions is investigated during wire drawing using finite element analysis and experiments. Based on a comparative study with *n* values of the wire with 0.0, 0.1, 0.5, and 1.0, the following conclusions were inferred:

1. The temperature increase of the wire augmented as the *n* of the specimen increased despite the same amount of ideal plastic deformation, which is associated closely with redundant work. The shear strain increased with the *n* of the specimen, and this shear strain generated redundant work, leading to a high temperature rise.
2. The drawing force increased with the *n* of the wire during wire drawing, which is related to the high redundant work with increasing *n* of the wire. In addition, the drawing force varied linearly with the temperature rise of the wire.
3. The damage value in the center region of the wire reduced with increasing *n* of the wire during wire drawing, because the effective stress increased with increasing the *n* of the wire
4. The drawing speed should be reduced and/or the cooling of wire and die should be strengthened during wire drawing with increasing *n* value of the wire, because product quality and die wear are highly associated with the temperature rise of the wire in the deformation zone.
5. From the results of the present study, engineers need to choose optimum drawing condition based on the variation of the drawing force and wire temperature with process conditions and materials. However, it is not easy to present quantitative suggestions for engineers based on the results of this study. Further research under more industrially specific conditions is required to design the optimum wire-drawing process with *n* value of material.

## Figures and Tables

**Table 1:** Operating conditions of wire-drawing test performed in present study.

Parameter	Value
Initial wire diameter (*d_o_*)	13.00 mm
Final wire diameter (*d_f_*)	11.63 mm
Reduction in area per pass (*R_p_*)	20%
Semi-die angle (*θ*)	6°
Drawing velocity (*V_d_*)	0.07 m/s

**Table 2:** Thermal properties of specimen and die used in present FEA.

Process Conditions	Wire Rod	Die
Thermal conductivity [*k*] (W/m/°C)	59 [37]	70 [38]
Heat capacity [*ρC_p_*] (N/mm^2^/°C)	3.6 [36]	3.6
Fraction factor (*ξ*)	0.9	-

**Table 3:** Flow stress of specimen used in current simulation.

Material	Flow Stress (MPa)
Non-hardening wire	*σ* = 391*ε*^0.0^
Low-hardening wire	*σ* = 500*ε*^0.1^
High-hardening wire	*σ* = 1250*ε*^0.5^
Linear-hardening wire	*σ* = 3554*ε*^1.0^

## Data Availability

Not applicable.
